# A Marine-Derived Anti-Inflammatory Scaffold for Accelerating Skin Repair in Diabetic Mice

**DOI:** 10.3390/md19090496

**Published:** 2021-08-30

**Authors:** Xiaoli Huang, Na Guan, Qiu Li

**Affiliations:** Central Laboratory and College of Chemistry and Pharmaceutical Sciences, Qingdao Agricultural University, Qingdao 266109, China; xiaolihuang@qau.edu.cn (X.H.); qingnong619@126.com (N.G.)

**Keywords:** brown alga polysaccharide, inflammatory, diabetic wound healing

## Abstract

Reconstructing the typical analogue of extracellular matrix (ECM) in engineered biomaterials is essential for promoting tissue repair. Here, we report an ECM-mimetic scaffold that successfully accelerated wound healing through enhancing vascularization and regulating inflammation. We prepared an electrospun fiber comprising a brown alga-derived polysaccharide (BAP) and polyvinyl alcohol (PVA). The two polymers in concert exerted the function upon the application of PVA/BAP2 fiber in vivo; it started to reduce the inflammation and promote angiogenesis at the wound site. Our serial in vitro and in vivo tests validated the efficacy of PVA/BAP2 fiber. Particularly, PVA/BAP2 fiber accelerated the repair of a full-thickness skin wound in diabetic mice and induced optimal neo-tissue formation. Generally, our results suggest that, by mimicking the function of ECM, this fiber as an engineered biomaterial can effectively promote the healing efficiency of diabetic wounds. Our investigation may inspire the development of new, effective, and safer marine-derived scaffold for tissue regeneration.

## 1. Introduction

The reconstruction of functional tissue and neovascularization is crucial for acute skin injuries such as massive trauma and diabetic ulcers [1,2,3,4]. Inflammatory response combined with tissue regeneration play a vital role in this comprehensive process, which is difficult to control and regulate [5,6]. For the normal wound, wound healing is well orchestrated and typically continues through the defined phases of hemostasis, inflammation, proliferation, and remodeling.

This process is disturbed in diabetic patients, marked by prolonged inflammation that is believed to defer wound healing [7]. At present, much research has been devoted to supplying anti-inflammatory treatments for this goal. However, the drugs are unable to be controlled precisely. Meanwhile, the safety of the exogenous drugs is also questioned. Recently, the issue of how to explore and develop the biomaterials for suppressing inflammatory response has received much increased attention. The ideal biomaterials can act as the cell matrixes that can modulate inflammation and support cell adhesion, proliferation, and so on. Much effort has been devoted to discovering suitable materials for this aim.

The brown alga, a type of seaweed, was found to be rich in polysaccharides, which is one of the more abundant algal resources in the sea [8,9]. Brown alga polysaccharide (BAP) has been proven to have a variety of pharmacological benefits, including anti-inflammation, antioxidant, hematopoietic, immune regulation, and gastrointestinal protection properties [10]. Nevertheless, it has been difficult to establish the ideal scaffold using the polysaccharide alone due to its solubility and viscosity. Polyvinyl alcohol (PVA), which is approved by the US Food and Drug Administration for numerous biomedical applications, can mix with the active polysaccharide [11] and provide good mechanical properties [12,13,14,15,16]. We tried to fabricate the mixed materials into the scaffold. Electrospinning is a versatile tool that has been applied for producing tissue engineering scaffolds [17,18]. This technology, which uses high-voltage electrostatic force to form the net structures, provides a biomimetic cellular matrix that possesses the nanoscale and microscale fibers for the cells interaction. Therefore, we choose PVA mixed with the polysaccharide co-electrospinning for fiber preparation.

**Figure 1:**
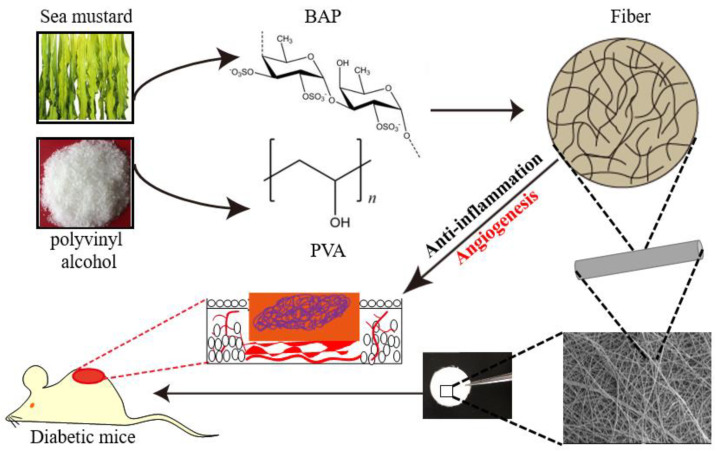
Schematic illustration of the designed ECM-mimetic fiber for wound healing in diabetic mice.

We expected that soon after scaffold implantation, PVA/BAP fiber starts to inhibit inflammation in a wound site. As healing continues, the fiber can promote tissue regeneration via enhancing angiogenesis (Figure 1). To validate our hypothesis, we first screened the PVA/BAP solutions at different ratios (from PVA/BAP 0–3) and employed the electrospinning technology to fabricate the solution into a fibrous scaffold, mimicking the microstructure of the skin tissue ECM. Then, we evaluated the anti-inflammation property of this scaffold in vitro. Further, we evaluated its effects on inhibiting inflammation and enhancing the function of promoting cell proliferation, inducing neovascularization, and eventually accelerating wound healing in diabetic mice with full-thickness wounds.

## 2. Results and Discussion

### 2.1. Preparation and Characterization of the Fibers

**Figure 2:**
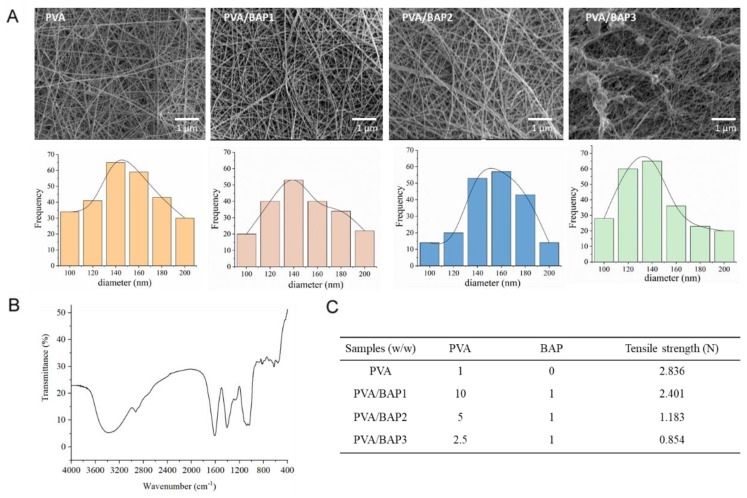
Characterization of nanofibers throughout the sample preparation. (**A**) The morphology of nanofibers loaded with BAP2 in different concentrations; (**B**) FT-IR measurement of BAP2; (**C**) tensile strength test of fibers loaded with BAP2 in different mass concentrations.

We prepared a series of fibers consisting of different concentrations of BAP polysaccharide. We optimized the proportion of PVA and BAP. The ratio of BAP and PVA was set from 1:10 to 1:2.5 (*w*/*w*). All the fibers added with polysaccharides had excellent physical and chemical properties. At first, the morphology of PVA/BAP fiber was shown to be smooth. However, with an increasing concentration of BAP, the PVA/BAP3 fiber was shown to be discontinuous according to the result of SEM (Figure 2A). In addition, SEM observation showed that the diameter of the fibers ranged between 120 and 180 nm.

We further confirmed the chemical structure of BAP using FT-IR technology. The absorption peak of -OH stretching vibration at 3419 cm^−1^ was shown. The peak of 1645 cm^−1^ was the -OH bending vibration absorption, and the peak of 1424 cm^−1^ was the absorption of CH_2_ deformation (Figure 2B). The mechanical property of a scaffold plays a vital role in affecting wound healing [19]. The tensile curves of fibers containing different concentrations of BAP are shown in Figure 2C. From the results, we can conclude that the tensile strength of nanofibers loaded with BAP was lower than pure PVA fiber. With the increasing proportion of BAP, the tensile strength of the fibers decreased. PVA/BAP2 encountered fracture at the elongation of 22%, and we suspected that the addition of BAP made the fibers more flexible.

### 2.2. The Water Absorption of the Fibers

**Figure 3:**
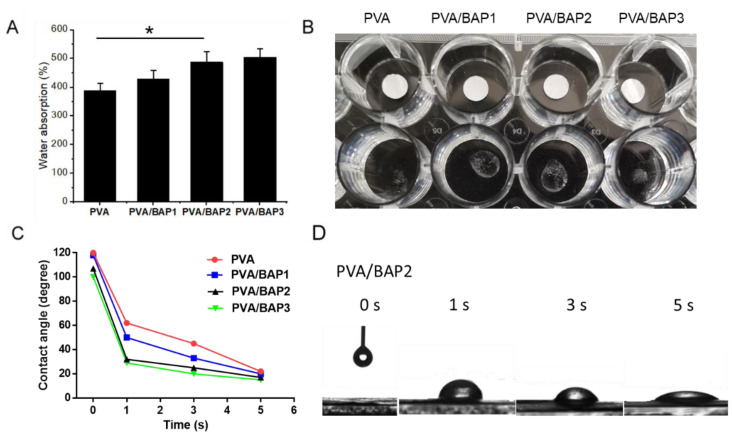
(**A**)Water absorption of nanofiber materials loaded with BAP of different mass concentrations (* *p* < 0.05; vs. PVA group); (**B**) the morphology change from fibrous scaffolds to sponges of different fibers after 1 min; (**C**) examination of the water absorption rate reflected by the change in instantaneous contact angle of the fibers; (**D**) the water contact angle of PVA/BAP2 at different time points.

We further characterized the water absorption properties of these fibers by the rate and capacity of water absorption and contact angle test. All the fibers absorbed water quickly and were fast to reach a swelling equilibrium. The lowest water absorption of about 380% was observed for the group of pure PVA, and it was nearly 500% for PVA/BAP2, suggesting that the swelling ratio increased with the increasing ratio of BAP in fibers (Figure 3A,B). In addition, all fibers were subjected to contact angle experiments to measure water absorption at 37 °C. The value of the water contact angle partly depends on the chemical components and the roughness of the interface materials [20]. The instant contact angle (ICA) of the fiber surface against a drop of water sharply decreased from 120 to almost 20 in 5 s (Figure 3C,D). According to the results, we can further confirm that the BAP improved the fiber permeability.

In sum, based on the above results, we can conclude that PVA/BAP2 fiber has homogenous morphology, high water absorption capacity, and a reasonable physical property for wound healing.

### 2.3. In Vitro Evaluation of the Fibers

**Figure 4:**
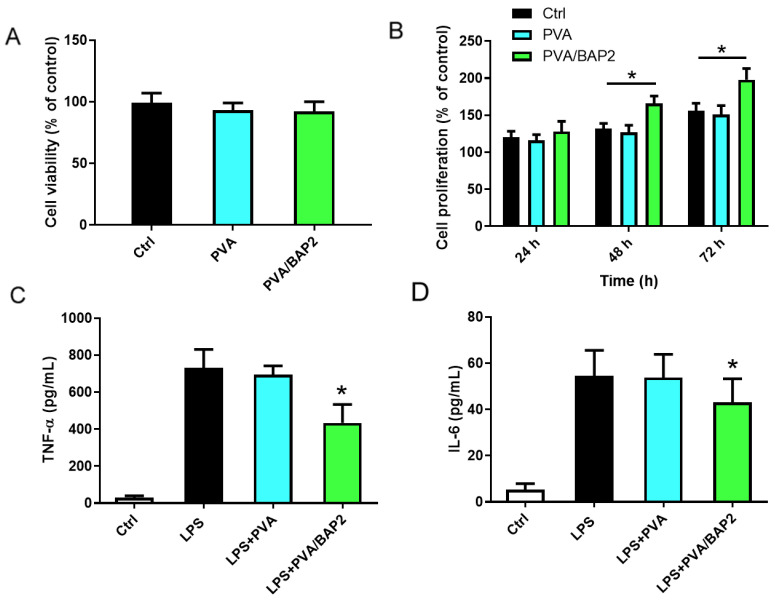
The in vitro evaluation of PVA/BAP2 fiber. (**A**) The effect on cell viability; (**B**) the effect on fibroblast proliferation; (**C**,**D**) the effect on cytokine levels of TNF-α and IL-6 in cell supernatant (* *p* < 0.05; vs. LPS group).

After we accomplished the physical characterizations, according to the above results, PVA/BAP2 was selected for further evaluation in vitro and in vivo. First, we set out to observe the effects of the fibers on cells. We examined the growth of macrophages on both PVA and PVA/BAP2 fibers, without or with BAP. The measurement of cell viability validated that both the fibers were non-toxic (Figure 4A). Further, we still did not know the fiber’s ability to support cell growth. Therefore, we observed the effect of this fiber on cell proliferation. Fibroblast cells, L929, were incubated over 72 h with or without BAP; the result showed that the cells proliferated faster on PVA/BAP2 fiber than on pure PVA (Figure 4B). Second, we tested whether the PVA/BAP2 fiber could affect the secretion of inflammatory factors such as TNF-α and IL-6. In this test, mouse macrophages—RAW 264.7 cells—were seeded onto fibers in a 24-well plate. Meanwhile, the cells as positive control were treated by LPS. After incubation in the plate for 12 h, the secretion of tumor necrosis factor (TNF-α) and interleukin 6 (IL-6) in the supernatant were measured by the corresponding ELISA kits, in accordance with the manufacturer’s instructions (4A BIOTECH, Beijing, China). The results indicate that the levels of TNF-α and IL-6, two major inflammatory cytokines, were markedly decreased in the PVA/BAP2 group, as compared with the LPS group (Figure 4C,D).

### 2.4. Rapid Repair of Wounds by the PVA/BAP2 Fiber

#### 2.4.1. The Fiber Accelerates Skin Repair in Diabetic Mice

**Figure 5:**
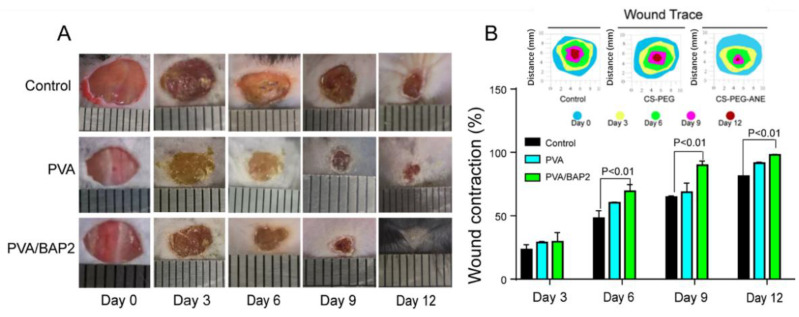
Healing process of diabetic wound treated by the different fibers. (**A**) Representative graphs of wound healing process of mice with different treatments; (**B**) Traces of wound-bed closure during 12 days for each treatment and wound closure rates at different time points of the different groups treated with the different fibers.

As demonstrated by the above results, PVA/BAP2 fiber exhibited multifunctional properties and evident an anti-inflammatory effect in vitro, which demonstrated a promising prospect for diabetic wound healing and tissue regeneration. As such, we examined the wound-healing ability of fibers for full-thickness wounds in mice. The wounds in diabetic mice were covered with the PVA fiber and PVA/BAP2 fiber. The untreated wounds in mice were set as a control group. As shown in Figure 5, the size of diabetic wounds was measured at 0, 3, 6, 9, and 12 days post-operation. The wound sizes measured at day 3 had no significant differences among the different groups; while from day 6, PVA/BAP2 fiber accelerated wound healing compared with other groups. On day 9, the wound healing rate of the fibers group facilitated faster healing than the control group. On day 12, the wound treated by PVA/BAP2 fiber exhibited the fastest healing rate, with complete closure and inconspicuous scar. However, wounds in other groups were still exposed and covered by eschar. Meanwhile, the quantitative analysis of wound healing rate was consistent with the gross morphology. On day 6, the wound contraction rate of the PVA/BAP2 group reached nearly 70%, which was significantly higher than the control group and the PVA group. After 12 days, the wounds closed completely in the PVA/BAP2 group, while wounds still had small scars in other groups (Figure 5A,B).

#### 2.4.2. Histomorphological Analysis of Skin Tissue

**Figure 6:**
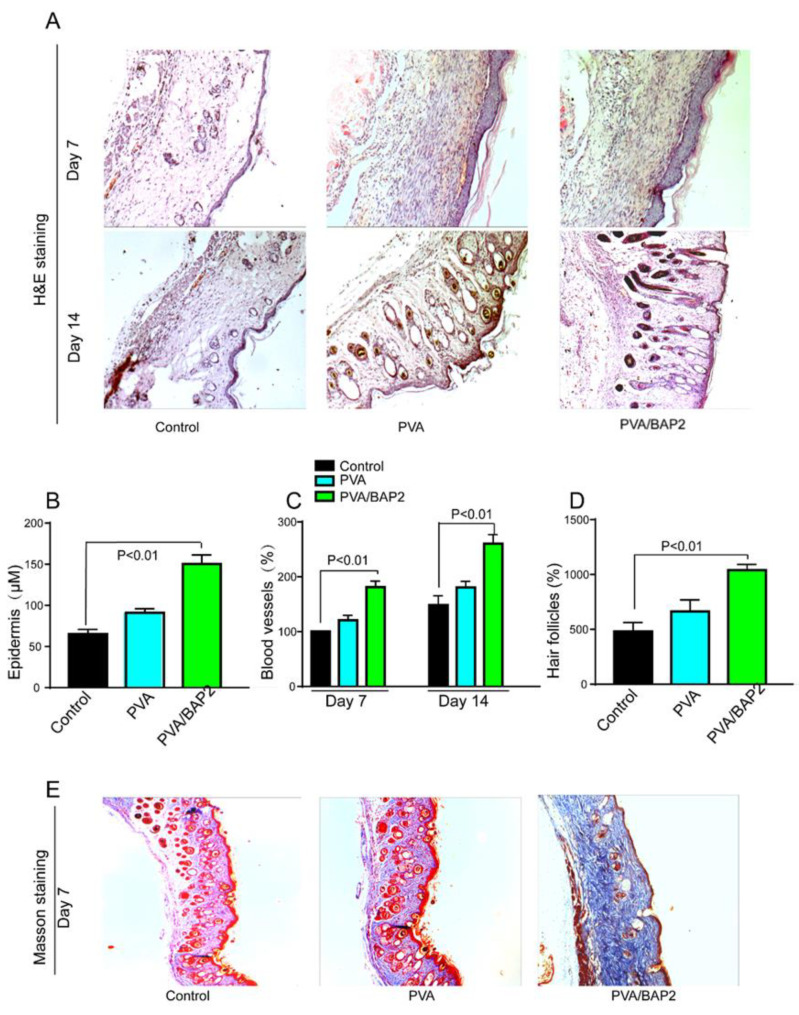
Histological evaluation of diabetic wound healing (amplification, ×10). (**A**) H&E staining of wounds at day 7 and 14; (**B**) The thickness of epidermis; (**C**,**D**) the number of blood vessels and hair follicles were measured; (**E**) the deposition of collagen fibers in the wound was stained at day 7.

The histological analysis was assessed first with H&E staining. From the results at day 7, we discovered that the epithelization and vascularization were accomplished better in PVA/BAP2-treated wounds than in other groups. At day 14, the wounds in all the groups were completely closed and gradually recovered to normal thickness (Figure 6A). Compared with the other two groups, we found there were more skin hair follicles and blood vessels in the PVA/BAP2 group. Meanwhile, wounds in PVA/BAP2 group showed obvious thickened epidermis compared with the control group (Figure 6B–D). We further performed Masson’s trichrome staining to observe the deposition of collagen in the wound tissue. It can be noted that the density of collagen deposition in the PVA/BAP2 group was much higher than that in the other groups, indicating that the addition of BAP2 facilitated collagen deposition (Figure 6E).

### 2.5. The Fibers Accelerate the Diabetic Wound Healing by Enhancement of Neovascularization

**Figure 7:**
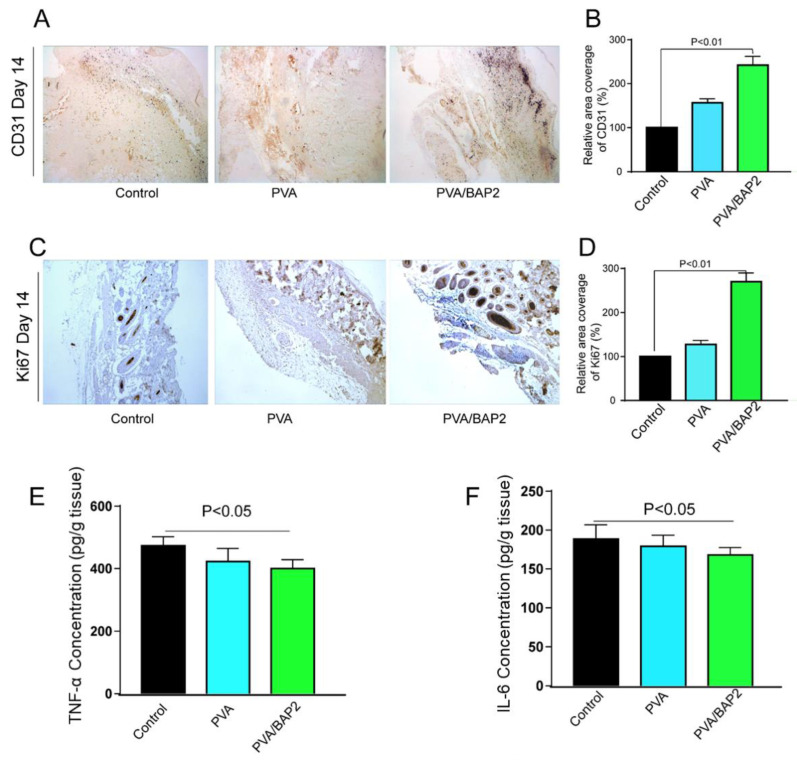
(**A**,**C**) Immunofluorescence staining of CD31 and Ki67 in skin wound tissues on day 14 (amplification, ×10) and quantitative analysis of the relative coverage area (**B**,**D**); Wound tissues were harvested at day 3 after different treatments, and the concentrations of chemokines TNF-α (**E**) and IL-6 (**F**) in wound tissues were determined by ELISA.

According to our design, the BAP2 in the fiber could play essential and versatile roles in the multiple stages of wound healing, of which two of the most likely roles are (i) to stimulate cell proliferation and (ii) to promote blood vessel formation. Therefore, we investigated whether this fiber could strengthen these two roles by examining the expression of ki-67 and CD31 markers at day 14 after implantation. From the results in Figure 7A–D, we can conclude that the PVA/BAP2 group showed the highest amount of Ki67 expression in all groups. Further, the addition of BAP2 promoted vessel regeneration, which was investigated by detecting the vessel endothelial cell marker CD31. Compared with the control group, the expression of CD31 was strongly enhanced by BAP2 in PVA/BAP2 group. The diabetic wound exists a persistent inflammatory phase and generates many inflammatory chemokines. The inflammatory chemokines at the wound site were detected by ELISA kits. As shown in Figure 7E,F, the concentrations of TNF-α and IL-6 in the group treated by PVA/BAP2 fiber were significantly decreased compared with other groups. The results suggest that PVA/BAP2 fiber could reduce the inflammatory response in the early stage of wound healing and could accelerate wound healing.

## 3. Materials and Methods

### 3.1. Materials

Brown algae polysaccharide (BAP, Purity 95%, MW 200–300 KDa) was purchased from Qingdao Seawin Biotech Group (Qingdao, China). The polysaccharide derived from sea mustard (a kind of brown algae) was prepared through defatting with 95% ethanol, water extract, and alcohol precipitate technology, in addition to filtration, concentration, dialysis with ultrafiltration, sterilization, and drying. Poly (vinyl alcohol) with a viscosity of 50–60 mpa-s, trifluoroethanol, and acetic acid (AcOH) were obtained from Sinopharm Group (Shanghai, China). All other chemicals and reagents at analytical grade were purchased from Sigma-Aldrich (St. Louis, MO, USA).

### 3.2. Electrospinning Fabrication of PVA/BAP Fibers

BAP/PVA fibers were prepared by electrospinning technology. Briefly, the PVA was dissolved in 50% acetic acid heated at 90 °C for 3 h and cooled to room temperature, then mixed with polysaccharides under vigorous stirring for 15 min before electrospinning. The two solutions were mixed to ensure the ratio of BAP to PVA to be 1:10, 1:5, and 1:2.5 (*w*/*w*), which were named as BAP1, BAP2, and BAP3, respectively. The mixture solution with PVA and BAP was filled into a 10 mL plastic syringe equipped with the electrospinning instrument (TongliWeina, Shenzhen, China). The fibers were collected by an aluminum foil collector. The voltage was set to 20 kV. The distance from the syringe to the collector was fixed at 15 cm, with a flow rate of 0.1 mL/h.

### 3.3. Characterization of the Fibers

The morphology of the fibers was observed by scanning electron microscope (JSM-7500F, Tokyo, Japan), with the average diameter calculated by Image J. The BAP was characterized using attenuated total reflectance Fourier transform infrared (ATR-FTIR) spectroscopy (JASCO FT/IR 620, Tokyo, Japan) over a range of 400–4000 cm^−1^. Furthermore, the mechanical tensile tests of fibers were assessed with TA Instruments equipped with linear tensile geometries [21]. The contact angle and surface tension were tested by contact angle measuring instrument at room temperature (KRUSS Cluis DSA25, Hamburg, Germany). The water absorption of wound dressing directly affects the wettability of the wound, and a moist environment is beneficial for wound healing [22]. The swelling rate of our fibers were evaluated in phosphate-buffered saline (PBS, pH = 7.4). Briefly, dry nanofibers were cut into small round pieces and weighed (w0). Then they were soaked in PBS and incubated at 37 °C and weighed (wt) at different time intervals until saturated. The swelling rate was calculated [23,24,25].

### 3.4. Cytotoxicity, Immunoregulatory Activity and Cell Growth In Vitro

The mouse macrophage—RAW 264.7 cell, obtained from the American Type Culture Collection (ATCC, Manassas, VA, USA)—was cultured in Dulbecco’s modified Eagle’s medium (DMEM) supplemented with 10% fetal bovine serum (FBS), 100 U/mL penicillin, and 100 mg/mL streptomycin in 5% CO2 at 37 °C, and it was selected for in vitro evaluation. RAW 264.7 cells (2 × 10^5^ cells/well) in 24-well plates pre-placed the different sterile fibers were incubated at 37 °C with 5% CO_2_ for 24 h. We added MTT into each well. After 4 h, each well was added to DMSO, and after another 1 h, the plate was vibrated for 5 min on a microplate fast oscillator. Subsequently, it was measured by a microplate reader instrument (MD SpectraMax M5, San Francisco, CA, USA). As described above, the cells were treated for 24 h. Then the cells were stimulated with LPS (1 μg/mL) for 18 h. Additionally, two major inflammatory cytokines—TNF-α and IL-6—were measured using ELISA kits according to manufacturer’s instruction (CME0004 and 0006, 4A Biotech Co., Ltd., Beijing, China).

The mouse fibroblast cell line L929, cultured in Dulbecco’s modified Eagle medium (DMEM) with the addition of 10% FBS, was selected for evaluation in vitro. The cells were seeded onto the fibers and incubated at 37 °C for 24 h. The cell viability was quantitatively measured using the MTT method at day 1, 3, and 5. MTT solution was added into each well, which was followed by incubation for 4 h at 37 °C. Then, 100 μL dimethyl sulfoxide (DMSO) was added to each well to dissolve formazan crystals. Finally, the samples were detected at 570 nm (Abs) using a microplate reader (MD SpectraMax M5, San Francisco, CA, USA).

### 3.5. Full-Thickness Wound Model

All animal protocols were approved by the Animal Care and Use Committee of Qingdao University and conformed to the Guidelines for the Care and Use of Laboratory Animals published by the National Institutes of Health (NIH Publication No. 8023, revised 1978). The experiments followed the regulations for the Care and Use of Laboratory Animals of the National Institute of Animal Health and the Guidance by the ethics committee of Qingdao University (animal welfare assurance number: 14-0027). The C57 mice (male, 18–20 g) were kept first for 24 h under the appropriate conditions. Then, the mice were injected with 1% streptozotocin (STZ, 65 mg/kg) to build a diabetic model. After that, the diabetic mice were anesthetized and one circular full-thickness skin wound (10 mm diameter) was cut off under sterile conditions. Then the diabetic-wound mice were randomly divided into four groups, including the control group, PVA group, BAP/PVA group (mice, *n* = 6). The mice were reared in different cages.

### 3.6. Gross View of Wound Healing

We observed the gross view of wounds at different time intervals after the operations. The wound healing rate was calculated according to comparison with the original area of wound. For each group, we cut 5 mm of surrounding intact tissue around the wound. The obtained tissues were immersed in paraformaldehyde (4%) for 24 h and then embedded in paraffin. After the paraffin was sectioned, hematoxylin-eosin (H&E) and Masson’s trichrome staining were performed for histological analysis.

### 3.7. Immunohistochemically (IHC) Staining of the Tissue Samples

The tissues in different groups were selected for immunohistochemical (IHC) staining. Briefly, the sections (3–5 μm) were placed on positively charged slides. The paraffin sections were dewaxed and rehydrated, and then the epitope was recovered in sodium citrate solution. After washing in phosphate-buffered saline with Tween 20 (PBST), anti-CD31 and anti-Ki67 (4A Biotechnology Co., Ltd., Beijing, China) were incubated with the tissues for 2 h at 37 °C, and the color was developed by DAB color reaction kit (ZSGB-BIO, Beijing, China). Finally, the paraffin sections were dyed by hematoxylin, dehydrated by alcohol, and sealed. The stained sections were observed, and all the images were captured on a microscope (Olympus, New York, NY, USA).

### 3.8. ELISAs for Cytokine Detection

The tissues around the wound were cut from diabetic mice at day 3 after the operation. Subsequently, the tissues were milled in cold phosphate-buffered saline (PBS) and then centrifuged at 12,000 rpm for 10 min at 4 °C to obtain the supernatants. After that, the concentrations of the cytokines including IL-6 and TNF-α were detected using the corresponding ELISA kits (4A Biotechnology Co., Ltd., Beijing, China).

### 3.9. Statistics

All data are presented as mean ± standard deviation (SD). Statistical analyses were performed using one-way ANOVA (GraphPad Prism 6), with * and ** standing for *p* < 0.05 and <0.01, respectively.

## 4. Conclusions

In this study, we report a fibrous scaffold based on marine-derived polysaccharide that successfully accelerated the healing of full-thickness skin wounds in diabetic mice. As its most prominent feature, PVA/BAP2 fiber not only owns good mechanical property but also has potent anti-inflammatory activity. Compared with the other general fibers, PVA/BAP2 fiber shortened the inflammatory period and prolonged the cell proliferation and reconstruction period. In sum, PVA/BAP2 fiber displayed great potential as a multifunctional wound dressings for the therapy of full-thickness diabetic wounds, which may represent a useful strategy for the design of regenerative materials.

## Data Availability

Not applicable.

## References

[1] Forbes S.J., Rosenthal N. (2014). Preparing the ground for tissue regeneration: From mechanism to therapy. Nat. Med..

[2] Thangavel P., Kannan R., Ramachandran B., Moorthy G., Suguna L., Muthuvijayan V. (2018). Development of reduced graphene oxide (rGO)-isabgol nanocomposite dressings for enhanced vascularization and accelerated wound healing in normal and diabetic rats. J. Colloid Interface Sci..

[3] Das S.K., Yuan Y.F., Li M.Q. (2017). An overview on current issues and challenges of endothelial progenitor cell-based neovascularization in patients with diabetic foot ulcer. Cell. Reprogr..

[4] Bourgine P.E., Gaudiello E., Pippenger B., Jaquiery C., Klein T., Pigeot S., Todorov A., Feliciano S., Banfi A., Martin I. (2017). Engineered extracellular matrices as biomaterials of tunable composition and function. Adv. Funct. Mater..

[5] Koh T.J., DiPietro L.A. (2011). Inflammation and wound healing: The role of the macrophage. Expert Rev. Mol. Med..

[6] Fahey T.J., Sadaty A., Jones W.G., Barber A., Smoller B., Shires G.T. (1991). Diabetes impairs the late inflammatory response to wound healing. J. Surg. Res..

[7] Brem H., Tomic-Canic M. (2007). Cellular and molecular basis of wound healing in diabetes. J. Clin. Investig..

[8] Michel G., Tonon T., Scornet D., Cock J.M., Kloareg B. (2010). The cell wall polysaccharide metabolism of the brown alga Ectocarpus siliculosus. Insights into the evolution of extracellular matrix polysaccharides in Eukaryotes. New Phytol..

[9] Wu G.-J., Shiu S.-M., Hsieh M.-C., Tsai G.-J. (2016). Anti-inflammatory activity of a sulfated polysaccharide from the brown alga Sargassum cristaefolium. Food Hydrocoll..

[10] Ananthi S., Raghavendran H.R.B., Sunil A.G., Gayathri V., Ramakrishnan G., Vasanthi H.R. (2010). In vitro antioxidant and in vivo anti-inflammatory potential of crude polysaccharide from Turbinaria ornata (Marine Brown Alga). Food Chem. Toxicol..

[11] Chen X., Lu B., Zhou D., Shao M., Xu W., Zhou Y. (2020). Photocrosslinking maleilated hyaluronate/methacrylated poly (vinyl alcohol) nanofibrous mats for hydrogel wound dressings. Int. J. Biol. Macromol..

[12] Allafchian A., Hosseini H., Ghoreishi S.M. (2020). Electrospinning of PVA-carboxymethyl cellulose nanofibers for flufenamic acid drug delivery. Int. J. Biol. Macromol..

[13] Baykara T., Taylan G. (2021). Coaxial electrospinning of PVA/Nigella seed oil nanofibers: Processing and morphological characterization. Mater. Sci. Eng. B.

[14] Bilginer R., Yildiz A.A. (2020). A facile method to fabricate propolis enriched biomimetic PVA architectures by co-electrospinning. Mater. Lett..

[15] Prahasti G., Zulfi A., Khairurrijal K. (2021). Synthesis of fiber membranes from polyvinyl alcohol (PVA)/shell extract of melinjo (SEM) using electrospinning method. Mater. Today Proc..

[16] Zhan F., Yan X., Li J., Sheng F., Li B. (2021). Encapsulation of tangeretin in PVA/PAA crosslinking electrospun fibers by emulsion-electrospinning: Morphology characterization, slow-release, and antioxidant activity assessment. Food Chem..

[17] Memic A., Abudula T., Mohammed H.S., Joshi Navare K., Colombani T., Bencherif S.A. (2019). Latest progress in electrospun nanofibers for wound healing applications. ACS Appl. Bio Mater..

[18] Zahedi E., Esmaeili A., Eslahi N., Shokrgozar M.A., Simchi A. (2019). Fabrication and characterization of Core-Shell electrospun fibrous Mats containing medicinal herbs for wound healing and skin tissue engineering. Marine Drugs.

[19] He T., Wang J., Huang P., Zeng B., Li H., Cao Q., Zhang S., Luo Z., Deng D.Y.B., Zhang H. (2015). Electrospinning polyvinylidene fluoride fibrous membranes containing anti-bacterial drugs used as wound dressing. Colloids Surf. B.

[20] Wang Z., Hu W., You W., Huang G., Tian W., Huselstein C., Wu C.-L., Xiao Y., Chen Y., Wang X. (2021). Antibacterial and angiogenic wound dressings for chronic persistent skin injury. Chem. Eng. J..

[21] Séon-Lutz M., Couffin A.-C., Vignoud S., Schlatter G., Hébraud A. (2019). Electrospinning in water and in situ crosslinking of hyaluronic acid / cyclodextrin nanofibers: Towards wound dressing with controlled drug release. Carbohydr. Polym..

[22] Qi L., Ou K., Hou Y., Yuan P., Yu W., Li X., Wang B., He J., Cui S., Chen X. (2021). Unidirectional water-transport antibacterial trilayered nanofiber-based wound dressings induced by hydrophilic-hydrophobic gradient and self-pumping effects. Mater. Des..

[23] Shahrousvand M., Haddadi-Asl V., Shahrousvand M. (2021). Step-by-step design of poly (ε-caprolactone) /chitosan/Melilotus officinalis extract electrospun nanofibers for wound dressing applications. Int. J. Biol. Macromol..

[24] Fang P.H., Kotkata M.F., Kandil K.M. (1987). Analysis of an electrical conductivity formula for an amorphous-crystalline selenium mixture. J. Non-Cryst. Solids.

[25] Perepechko I.I. (1974). On the applicability of the Mosley formula to determine the orientation of crystalline polymers. Polymer Sci. USSR.

